# Novel Insights into Mercury Effects on Hemoglobin and Membrane Proteins in Human Erythrocytes

**DOI:** 10.3390/molecules25143278

**Published:** 2020-07-19

**Authors:** Marina Piscopo, Rosaria Notariale, Fabiana Tortora, Gennaro Lettieri, Giancarlo Palumbo, Caterina Manna

**Affiliations:** 1 Department of Biology, University of Naples Federico II, 80126 Naples, Italy; gennarole@outlook.com; 2 Department of Precision Medicine, School of Medicine, University of Campania “Luigi Vanvitelli”, via Luigi de Crecchio, 80138 Naples, Italy; notarialer@gmail.com (R.N.); fabiana.tortora1989@libero.it (F.T.); 3 Department of Economics, Management, Institutions, University of Naples Federico II, via Cupa Nuova Cinthia, 80126 Naples, Italy; gpalumbo@unina.it

**Keywords:** mercury, red blood cell, hemoglobin, membrane proteins, band 3 protein, cytoskeleton, thiols, protein modifications, metal–ion interaction

## Abstract

Mercury (Hg) is a global environmental pollutant that affects human and ecosystem health. With the aim of exploring the Hg-induced protein modifications, intact human erythrocytes were exposed to HgCl_2_ (1–60 µM) and cytosolic and membrane proteins were analyzed by SDS-PAGE and AU-PAGE. A spectrofluorimetric assay for quantification of Reactive Oxygen Species (ROS) generation was also performed. Hg^2+^ exposure induces alterations in the electrophoretic profile of cytosolic proteins with a significant decrease in the intensity of the hemoglobin monomer, associated with the appearance of a 64 kDa band, identified as a mercurized tetrameric form. This protein decreases with increasing HgCl_2_ concentrations and Hg-induced ROS formation. Moreover, it appears resistant to urea denaturation and it is only partially dissociated by exposure to dithiothreitol, likely due to additional protein–Hg interactions involved in aggregate formation. In addition, specific membrane proteins, including band 3 and cytoskeletal proteins 4.1 and 4.2, are affected by Hg^2+^-treatment. The findings reported provide new insights into the Hg-induced possible detrimental effects on erythrocyte physiology, mainly related to alterations in the oxygen binding capacity of hemoglobin as well as decreases in band 3-mediated anion exchange. Finally, modifications of cytoskeletal proteins 4.1 and 4.2 could contribute to the previously reported alteration in cell morphology.

## 1. Introduction

Human exposure to heavy metals has increased significantly in the last decades as a result of a parallel increment in the use of these metals in industrial processes [1] and products [2,3]. Therefore, considerable attention has been focused on environmental pollution and the biological effects of these elements [4,5], including mercury (Hg) [6]. Hg contamination is extensive in all environmental compartments such as soil, air and water [7,8] and human exposure to Hg has increased with modern industrialization due to its anthropogenic emissions from fuel combustion, municipal incinerators and chemical industries. The pathways of Hg exposure in the general population mainly include respiration and the ingestion of food [9].

The health consequences of such exposure can be severe, leading to adverse health outcomes [10,11,12,13]. Renal damage [14,15] and neuronal disorders [16,17] are well characterized toxic effects of Hg. The contribution of environmental pollutants, including Hg, to the etiology of autism spectrum disorder has been recently reviewed [18]. In addition, prolonged Hg exposure is considered as a contributing risk factor for Cardiovascular Disease [19,20]. In this respect, a recent meta-analysis indicates a significant positive association between high Hg body burden and hypertension, likely due to both endothelial and renal dysfunction [21]. Furthermore, Wierzbicki et al. [22] reported that workers occupationally exposed to mercuric vapors exhibited a statistically significant increase in blood coagulation along with increased thrombin generation. Interestingly, in vitro exposure of intact red blood cell (RBC) to mercuric chloride (HgCl_2_) induces morphological changes [23], which increase their pro-coagulant activity [24]. RBC are indeed an important target of Hg toxicity because this metal preferentially accumulates in these cells [25]. The literature data on the mechanisms involved in the transport of mercuric ions in target tissue have recently been reviewed by Bridges and Zalups [26]. As far as mercury uptake by RBC is concerned, the majority of the data refers to organic forms of mercury, indicating that when rats are injected with a nonnephrotoxic dose of methyl mercury, approximately 30% of the administered dose is detected in blood after 24 h and 99% of blood Hg is associated with RBC [27]. The study of HgCl_2_ uptake by RBC has been addressed by a few old papers, which report that RBC uptake of HgCl_2_ is rapid and reaches equilibrium concentration in a few minutes [28].

Heavy metals taken into the organism can exert their toxic effects through different mechanisms [29,30]. As far as Hg toxicity is concerned, it is endowed with a high affinity for sulphydryl groups; therefore this metal is able to react with small molecular-weight (MW) thiols, including glutathione (GSH), thus impairing the antioxidant defence system [31,32]. In addition, by reacting with crucial cysteine (Cys) residues, Hg may interact with cellular proteins, thus altering and inhibiting their enzymatic and structural functions and potentially leading to severe dysfunction in cellular activities [33]. In particular, Hg inhibitory activity on individual glycolytic enzymes, such as hexokinase and phosphofructokinase, has been reported [34]. Important mitochondrial functions are also affected by Hg, such as the activity of F_1_-F_0_-ATPase [35]. Furthermore, carnitine/acylcarnitine transporter has been identified as a possible target of Hg toxicity [36].

To further investigate Hg-induced cytotoxicity at the molecular level, the possible interaction of this heavy metal with RBC proteins was studied, using a cellular model system. In this paper, we offer experimental evidence that both hemoglobin (Hb) as well as specific membrane proteins can be altered by HgCl_2_ treatment, as revealed by sodium dodecyl sulfate-polyacrylamide gel electrophoresis (SDS-PAGE) and Acetic Acid Urea-Polyacrylamide Gel Electrophoresis (AU-PAGE). To the best of our knowledge, no studies have been published using this experimental approach. A discussion on the possible Hg^2+^–protein chemical interactions and correlation between reactive oxygen species (ROS) production and Hb aggregation state is also provided.

## 2. Results

In order to explore the possible interaction of Hg with erythrocyte proteins, intact human RBC were exposed for 4 h in vitro to increasing HgCl_2_ concentrations (1–60 µM) and the electrophoretic profiles of both cytosolic and membrane proteins were evaluated. In our previous studies, exploring the dose-dependency of Hg toxic effects on RBC, this HgCl_2_ concentration range was found to be optimal in order to study the oxidative stress (OS)-mediated cytotoxicity in these cells [37,38,39]

### 2.1. Electrophoretic Analyses of Cytosolic Proteins from Hg-Exposed Human RBC

**Figure 1:**
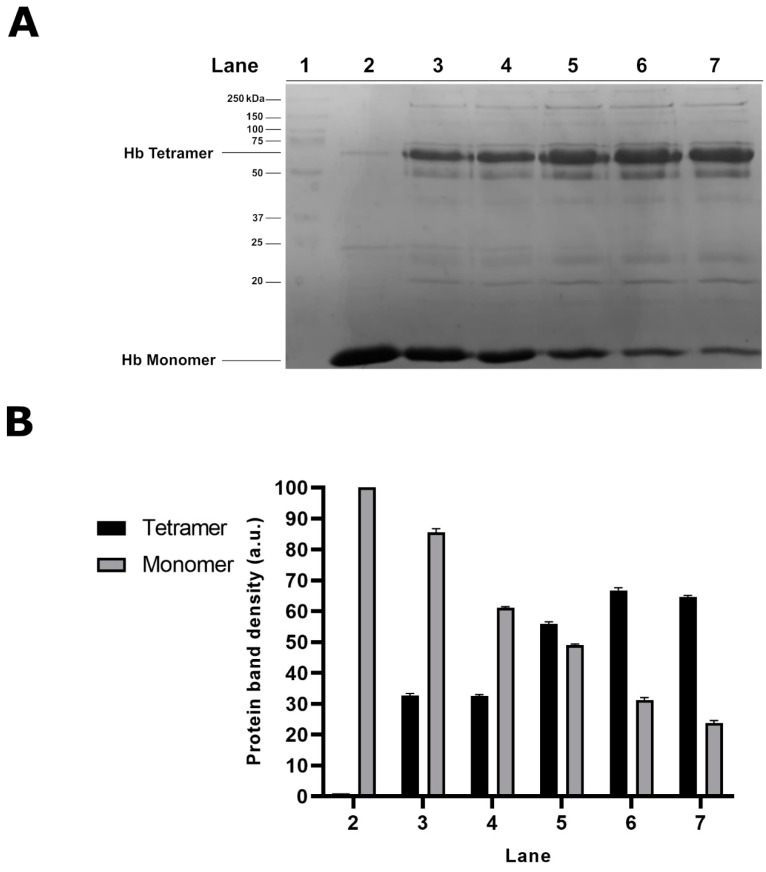
Sodium dodecyl sulfate-polyacrylamide gel electrophoresis (SDS-PAGE) analysis of the cytosolic fraction from mercury (Hg)-treated human red blood cell (RBC). Intact RBC were exposed in vitro for 4 h to different concentrations of mercuric chloride (HgCl_2_) and the cytosolic fractions analysed by SDS-PAGE. (**A**) Electrophoretic profile of the cytosolic fractions from untreated RBC (lane 2) and cells exposed to 60, 40, 20, 10 and 1 µM HgCl_2_ (lanes 3–7, respectively). Molecular weight (MW) markers (lane 1). (**B**) Densitometric analysis of the 16 and 64 kDa protein bands. The values shown are relative to n = 5 repeats.

As shown in Figure 1A (lane 2), the SDS-PAGE of the cytosolic fraction from control cells reveals the presence of a main band, identified as the Hb monomer according to its MW. Exposure of cells to all tested HgCl_2_ concentrations results in a significant alteration in the electrophoretic profile of the cytosolic proteins, with the appearance of additional protein bands with reduced mobility compared to the Hb monomer, likely indicating the formation of aggregates.

Among them, the most representative reduced mobility protein band, showing an apparent MW of 64 kDa, might represent a tetrameric form of Hb. Accordingly, the densitometric analysis of the two main protein bands (16 and 64 kDa), shown in Figure 1B, clearly indicated that, following the treatment of RBC with HgCl_2_, the 64 kDa protein band is formed at the expense of the Hb monomer. In addition, it is worth noting that the intensity of the 64 kDa protein band is maximum in samples from cells exposed to 1 µM, while it decreases with increasing HgCl_2_ concentrations.

**Figure 2:**
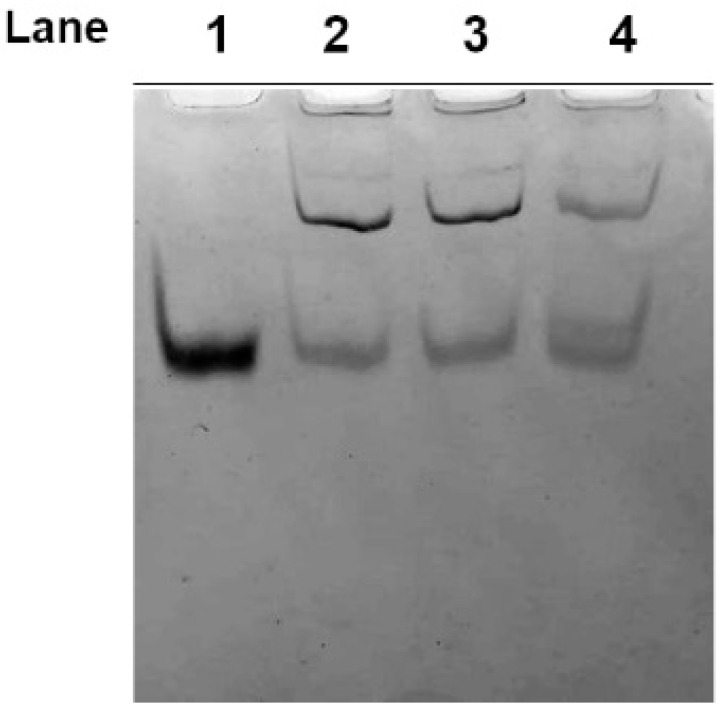
Acetic Acid Urea-Polyacrylamide Gel Electrophoresis (AU-PAGE) analysis of the cytosolic fraction from Hg-treated human RBC. Intact RBC were exposed in vitro for 4 h to different concentrations of HgCl_2_ and the cytosolic fraction analysed by AU-PAGE. Samples from untreated RBC (lane 1) and cells exposed to 1, 10 and 60 µM HgCl_2_ (lanes 2–4, respectively).

The formation of the Hg-induced protein aggregates was also confirmed by AU-PAGE analysis of samples obtained from cells exposed to 1, 10 and 60 µM HgCl_2_, also indicating that these Hg-induced protein aggregates are resistant to urea denaturation (Figure 2).

### 2.2. Effect of Dithiothreitol on Hg-Induced Protein Aggregate Formation in Human RBC

**Figure 3:**
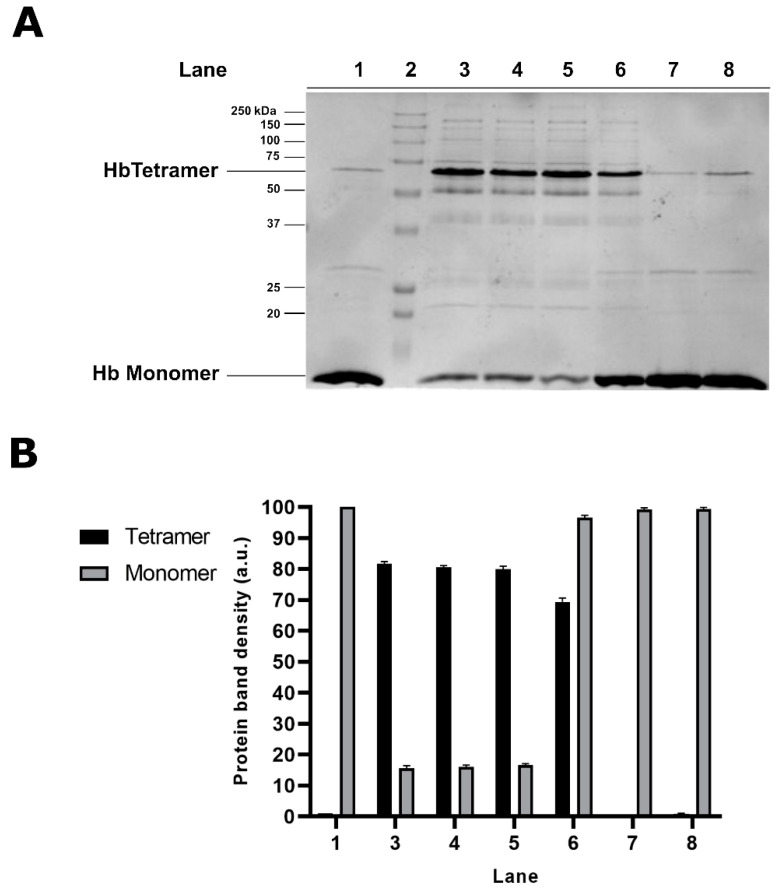
Effect of Dithiothreitol (DTT) on Hg-induced protein aggregates in human RBC. Intact RBC were treated with for 30 min with 30 mM DTT before and after cell exposure to with 1 µM HgCl_2_ and the cytosolic fractions analysed by SDS-PAGE. (**A**) Electrophoretic profile of samples from untreated RBC (lane 1) and from cells exposed to 1 µM HgCl_2_ (lanes 3–5); MW markers (lane 2); samples from RBC treated with HgCl_2_ and then with DTT (lane 6); Hb from RBC treated only with DTT (lane 7); Hb from RBC treated with DTT and then with HgCl_2_ (lane 8). (**B**) Densitometric analysis of the 16 and 64 kDa protein bands. The values shown are relative to n = 5 repeats.

In order to investigate the possible involvement of sulfhydryl groups in the formation of protein aggregates in Hg^2+^-exposed cells, the effect of Dithiothreitol (DTT) was tested, adding the reduced agent either prior or subsequent to cell treatment with HgCl_2_. The analysis by SDS-PAGE of the cytosolic fraction of RBC exposed to 1 µM HgCl_2_, followed by DTT treatment for 30 min, reveals a significant decrease in the intensity of the band corresponding to tetrameric Hb, associated with a parallel increase in the Hb monomer, as shown in Figure 3A (lane 6). This finding indicates that DTT treatment causes a partial dissociation of the aggregates, probably reducing sulphur-related bonds. Interestingly, longer DTT cell treatment (60 and 120 min) does not result in the complete disappearance of the 64 kDa protein band (data not shown), likely due to additional protein–Hg interactions involved in protein aggregate formation. Conversely, RBC pre-treatment with DTT, followed by cell exposure to HgCl_2_, prevents the Hg^2+^-induced modification of the electrophoretic profile completely. In this experimental condition, indeed, we can hypothesize that DTT may react rapidly and directly with the heavy metal, thus interfering with the interactions between Hg^2+^ and protein.

### 2.3. Electrophoretic Analyses of Membrane Proteins from Hg-Exposed Human RBC

**Figure 4:**
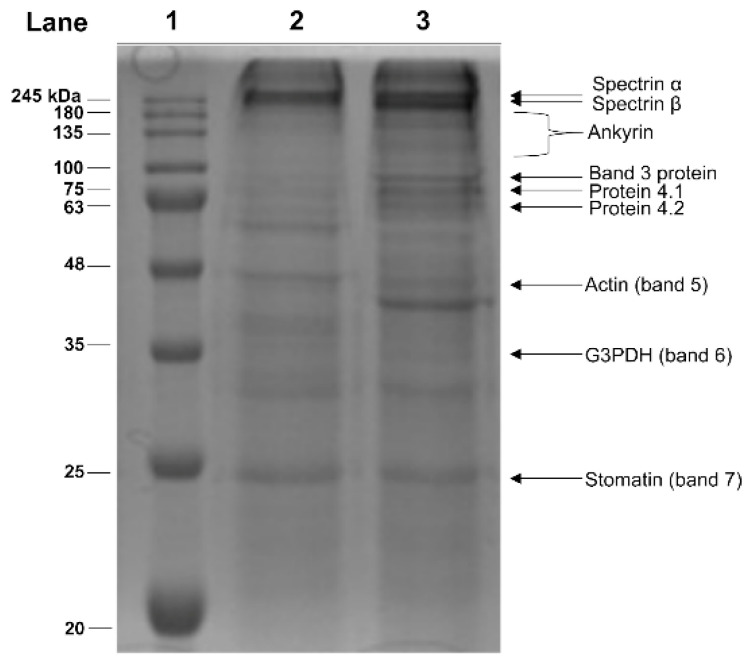
SDS-PAGE analysis of the membrane fraction from Hg-treated human RBC. Intact RBC were exposed in vitro for 4 h to 1 µM HgCl_2_ and the membrane fractions analysed by SDS-PAGE. MW markers (lane 1); sample from untreated (lane 2) and Hg-treated RBC (lane 3).

On the basis of these results, we use 1 µM HgCl_2_ to evaluate the effect of this heavy metal on the erythrocyte membrane proteins. The cellular membrane is a major target of Hg damage in RBC, particularly related to phosphatidylserine translocation to the cellular surface, which implies loss of membrane asymmetry [40]. Moreover, in our previous paper, an overall decrease in erythrocyte membrane protein thiols has been reported [23]. Therefore, with the aim to identify the specific Hg^2+^-modified erythrocyte membrane proteins, alterations in the electrophoretic profile of the membrane fraction from Hg^2+^-exposed RBC was analysed by SDS-PAGE. The typical electrophoretic profile of RBC membrane proteins is shown in Figure 4. The Hg^2+^ treatment of RBC results in the appearance of an additional protein band of apparent MW of about 40 kDa, undetectable in the control RBC. Moreover, in Hg^2+^-treated samples, difference in the protein band ratios is evident. In particular, an increase in the intensity of bands 3, 4.1, 4.2 and a decrease in the intensity of the band corresponding to glyceraldehyde-3-phosphate-dehydrogenase are observable.

### 2.4. Effect of Hg on ROS Formation in Human RBC

**Figure 5:**
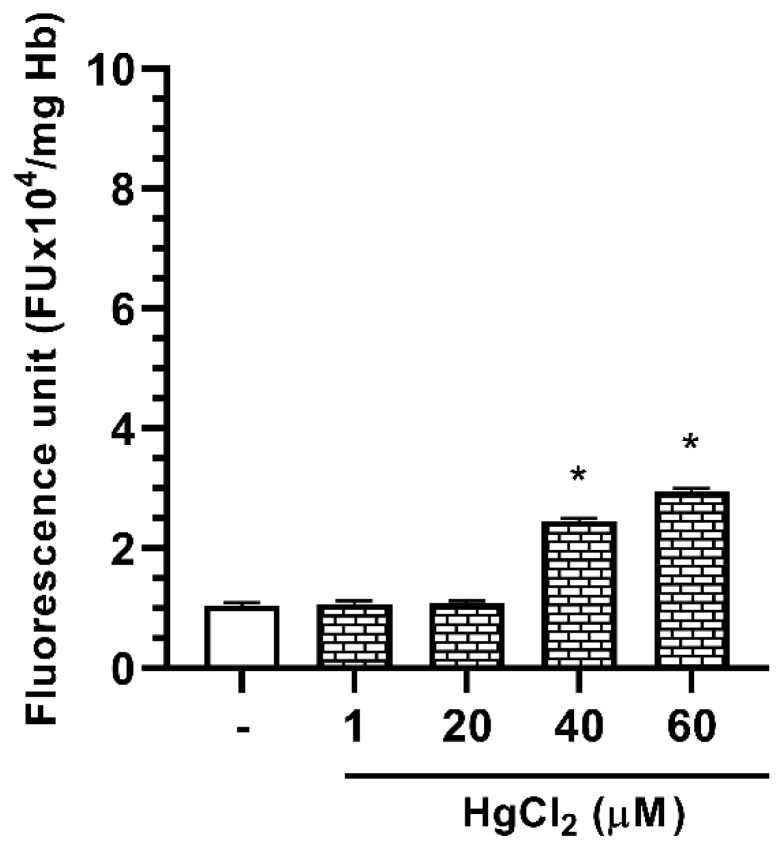
Effect of HgCl_2_ treatment on reactive oxygen species (ROS) production in RBC. Intact RBC were exposed in vitro for 4 h to different concentrations of HgCl_2_ and ROS production was evaluated by means of the fluorescent probe Dichlorofluorescein (DCF). Data are the means ± SEM (n = 9). Statistical analysis was performed with one-way ANOVA followed by Dunnett’s test (* *p* < 0.05).

In order to investigate the possible role of the OS in the Hg-induced erythrocyte protein modifications, the Dichlorofluorescein (DCF) fluorescent assay was performed to measure ROS formation. As shown in Figure 5, in our experimental conditions, RBC incubation in the presence of 1–60 µM HgCl_2_ results in an increase in DCF fluorescent signal, thus indicating a dose-dependent ROS formation, starting from RBC samples exposed to 40 µM HgCl_2_. This finding suggests that HgCl_2_ treatment determines the exposition of RBC to an oxidative microenvironment only at the higher metal concentrations. Interestingly, no Hg^2+^-induced ROS formation occurs in the concentration range 1–20 µM, in which the maximum effect of Hg^2+^ on protein aggregation is observable. 

## 3. Discussion

In recent decades, heavy metals—widespread environmental pollutants—received considerable scientific attention because their potential health and environmental risks [1,2,41]. The toxic biological effects of human exposure to these metals are extremely numerous. Among the molecular mechanisms underlying their toxicity, protein interactions seem to play a key role, possibly leading to structural and functional alterations, thus interfering with important metabolic as well as regulatory cellular function [33,42,43,44].

Here, we report data on the Hg^2+^-induced RBC protein alterations, as revealed by the electrophoretic analysis of both cytosolic as well as membrane fractions. Blood represents a major target of toxicants that enter the body through any route and RBC are particularly vulnerable to their harmful effects [25], being a preferential store for toxic heavy metals. In particular, Hg accumulates in these cells, mainly bound to the SH group of the cellular thiol GSH, present in very high concentrations in these cells [45].

In this study, we exposed intact human RBC to HgCl_2_ in vitro in the range of 1–60 μM and we demonstrated that this treatment induces significant alterations in the electrophoretic behaviour of both Hb and membrane proteins. As far as the Hg–Hb interaction is concerned, the presence of protein bands with reduced mobility compared to that corresponding to the Hb monomer was observed throughout the range of concentrations utilized. In particular, a protein band likely corresponding to a Hb tetramer on the basis of its apparent MW was observable. These data are in agreement with the reported Hg-induced protein coagulation effect on purified bovine Hb, incubated in vitro in the presence of mercuric acetate [46]. At this stage of investigation, we do not provide direct evidence of increased or decreased oxygen affinity for mercurized-Hb. However, if we assume that the Hg binding sites [46] should be likely located at or near the dimer–dimer contact interface, we would expect rather strong Hg-induced alterations in Hb monomers interactions, affecting the physiological cooperative conformational changes necessary for the oxygenation/deoxygenation process. Furthermore, the possibility that such Hg-induced perturbations would lead to the autooxidation of the ferrous iron of the heme to form methemoglobin can not be ruled out, resulting in the loss of ability to bind oxygen. Particularly interesting is the finding that Hg^2+^-induced Hb polymer formation is observed starting from a concentration as low as 1 μM. It is interesting to note, in this respect, that similar Hg concentrations have been found in the blood of individuals exposed to specific working environments, such as gold mines, as well as in people living in the surrounding areas [47]. Furthermore, workers occupationally exposed to Hg vapor show increased Hg blood concentration up to 0.4 µM, associated with significant alteration in the coagulation system [22]. Finally, in a recent paper, Forte et al. [48] reported abnormally high blood Hg levels in people living close to contaminated areas in Southern Italy. Increased Hg blood level is also strongly related to contaminated fish consumption [49].

An unexpected figure in the reported findings is that the amount of “mercurized” tetrameric Hb form decreases with increasing HgCl_2_ concentrations. A possible explanation of the inversely proportional dose-dependence of the Hg^2+^-induced polymer formation is that it may be related to OS. This element is a powerful but indirect inducer of OS in biological systems, as reported in our previous studies, in which the experimental evidence indicates that Hg^2+^-induced ROS generation is a late event in RBC, subsequent to a significant decrease in GSH [40]. This has recently been confirmed, in similar experimental conditions, by Ahmad and Mahmood [25] also for reactive nitrogen species formation. Therefore, increased OS may cause, at the higher concentrations utilized in our study, alterations in Hb aminoacidic residues incompatible or less compatible with the tetrameric form. 

Concerning the possible specific amino acid residues that are able to interact with Hg^2+^, our data on the significant reduction in the tetrameric form upon incubation of the Hg^2+^-treated RBC with the reducing agent DTT allow us to hypothesize that accessible Cys residues represent the preferential site of Hg–protein interaction. It is worth noting, in this respect, that two critical Cys in position 93 of the beta-chains have been identified as nitric oxide (NO) ligand, playing a role in Hb-mediated NO release [50]. Thus, the alteration of Cys-93, following Hg^2+^ interaction, might impair the Hb-mediated regulation of blood flow, therefore representing one of the physiologically important manifestations of Hg poisoning in these cells. Additional Hg-binding sites cannot be excluded, likely enhancing the aggregation process, including histidine residues. In this respect, the Hg-induced chymotrypsin aggregate formation is reported to be mediated by Hg^2+^–histidine interaction [51].

In addition, the effect of Hg^2+^ on RBC membrane proteins was also evaluated. A significant depletion in the overall membrane thiols has been reported in our previous paper, in similar experimental conditions [23]. The SDS-PAGE analysis revealed that cell treatment with HgCl_2_ results in alterations in the membrane protein electrophoretic profile, particularly related to bands 3, 4.1 and 4.2. Band 3 protein (B3p) is the most abundant integral protein on the RBC membrane, endowed with specific anion exchange capabilities, being responsible for acid balance, ion distribution and gas exchange [52,53]. Furthermore, it is also involved in RBC mechanical and osmotic properties, such as maintenance of biconcave cell shape and deformability. In particular, B3p is involved in specific protein–protein interactions, by connecting plasma membrane to the underlying cytoskeleton, through SH groups of specific Cys residues [53]. In this respect, Cys-201 and/or Cys-317, located at the cytoplasmic domain of B3p, are critical for ankyrin binding [54]. Accordingly, their derivatization with thiol reagents are reported to block ankyrin interaction and a specific antibody raised against a synthetic peptide containing Cys-201 acts as a potent inhibitor of ankyrin binding [55]. Moreover, band 4.1 and 4.2 proteins, key components of the cytoskeleton network [56], also contain accessible cysteines [57,58]. In conclusion, a large body of literature, accumulated over several years by our group and others, clearly demonstrates the RBC structural and metabolic alterations induced by Hg, indicating severe alterations in the physiology and morphology of these cells. Among these, exposure of phosphatidylserine at the level of the membrane is the most serious and most studied [40]. Taken together, the findings reported in this paper provide new insight in the Hg-induced alterations in human RBC using a cellular model system on hemoglobin and membrane proteins. Concerning the latter, our work represents the first study revealing alterations of specific membrane proteins, including those of the cytoskeleton, partially responsible for the morphological changes that are reported in our previous studies, consequent to exposure of intact cells in the presence of HgCl_2_ [23]. In particular, the observed structural modification to band 3 could suggest an alteration in the RBC anionic exchange capability that deserves further investigations.

## 4. Materials and Methods

### 4.1. Preparation of RBC and HgCl_2_ Treatment

Whole blood was taken by venipuncture from healthy volunteers, collected in heparinized tubes and centrifuged at 1200× *g* for 15 min at 4 °C. After removal of the buffy coat, the RBC fraction was washed twice with isotonic saline solution (0.9% NaCl) and resuspended in Krebs buffer (NaCl 7.305 g/L; KCl 0.79 g/L; MgSO_4_ 0.29 g/L; Hepes 7.69 g/L; CaCl_2_ 0.11 g/L; NaOH 0.54 g/L, containing 2.8 mM glucose) to obtain a 10% (*v*/*v*) hematocrit. Hg treatment was performed by incubation of aliquots of the cell suspension at 37 °C for 4 h, in the presence of different concentrations of HgCl_2_ in the range of 1–60 µM. A stock solution of HgCl_2_ was diluted in Krebs buffer.

### 4.2. Dithiothreitol (DTT) Treatment

The effect of DTT was tested by adding the reduced agent, at a final concentration of 30 mM, 30 min prior or subsequent to RBC incubation for 4 h with 1 µM HgCl_2_. Cell treatment with DTT after HgCl_2_ incubation was also repeated at 60 and 120 min. 

### 4.3. Preparation of Erythrocyte Membranes

The erythrocyte membrane fractions were prepared according to Galletti et al. [59], with minor modifications. After Hg^2+^ treatment, the samples were centrifuged and the hemolysis was performed by rapidly mixing packed RBC with 40 volumes of 5 mM sodium phosphate, pH 8.8. The membrane pellets, obtained by centrifugation at 27,000× *g* for 30 min at 4 °C, were then washed several times with the lysis buffer to remove unspecifically bound Hb. Creamy white ghosts were resuspended in 0.2% SDS and incubated for 30 min at 37 °C, prior to being used for the electrophoretic analysis.

### 4.4. Electrophoretic Analyses 

SDS-PAGE for cytosolic fraction proteins was performed as previously described in Vassalli et al. [60]. For membrane proteins, a stacking gel at 4.0% (*w*/*v*) acrylamide (acrylamide/bis-acrylamide 25:0.67) and a separating gel at 12.0% (*w*/*v*) acrylamide (acrylamide/bis-acrylamide 25:0.67) was utilized. At the end of the run, the gels were stained with Coomassie Brilliant Blue and acquired using a GelDoc system via Quantity One v.4.4.0 software (BioRad, Hercules, CA, USA). The densitometric analysis of gel bands was carried out using the software ImageJ.

AU-PAGE was performed as previously described in Fioretti et al. [61] with modifications reported in Piscopo et al. [62]. Protein samples were run on polyacrylamide gels prepared at 9% (*w*/*v*) acrylamide (acrylamide:bisacrylamide 25:0.67). The gels were stained with Amido Black.

### 4.5. Determination of Reactive Oxygen Species (ROS)

Dichlorofluorescein (DCF) assay was performed to detect ROS generation, according to Tagliafierro et al. Intact RBC were incubated with the nonpolar, non-fluorescent 2′,7′-dichlorodihydrofluorescin diacetate (DCFH-DA, Sigma-Aldrich, St. Louis, MO, USA), at a final concentration of 10 μM for 15 min at 37 °C. After centrifugation of cellular suspension at 1200× *g* for 5 min, the supernatant was removed, and the hematocrit was adjusted to 10% with Krebs, and RBC were treated with the selected HgCl_2_ concentrations in the dark for 4 h at 37 °C. At the end of the treatment, 20 μL of RBC suspension was diluted in 2 mL of distilled water and the fluorescent intensity of the oxidative derivative DCF was recorded (λ_exc_ 502; λ_em_ 520). The results were expressed as fluorescent intensity/mg Hb.

### 4.6. Protein Determination

Protein concentration was determined by the method of Bradford [63], using bovine serum albumin as standard.

### 4.7. Statistical Analyses

Images in figures are representative of 4–6 independent experiments performed with RBC of different donors. Data of ROS evaluation were expressed as means ± S.E.M of 3 independent experiments performed with different donors. The significance of differences was determined by one-way ANOVA followed by a post hoc Dunnett’s multiple comparisons test, using p < 0.05 as the criterion of significance. GraphPad Prism 8 was utilized for statistical analysis.
